# Supplementary material for: Pressure for rapid and accurate mate recognition promotes avian‐perceived plumage sexual dichromatism in true thrushes (genus: Turdus)
Source: J Evol Biol. 2022 Oct 5;35(11):1558–67. doi: 10.1111/jeb.14089 (PMC9828161; doi:10.1111/jeb.14089)
Supplement: Supplementary file 1 — Appendix S1 [file JEB-35-1558-s001.docx]

Supplementary Material: Rapid mate recognition promotes greater avian-perceived plumage sexual dichromatism in true thrushes (genus: *Turdus*)

## Supplementary Tables and Figures


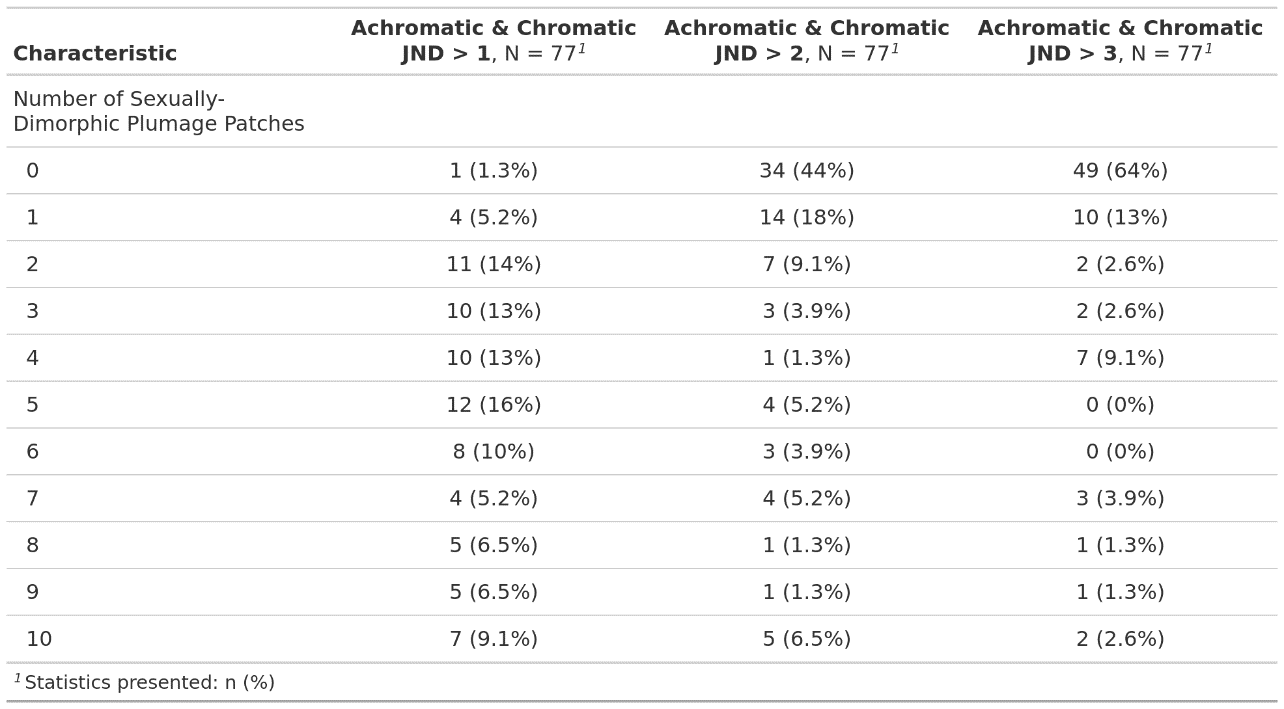


**Table S1**: Number of sexually-dimorphic plumage patches for combined achromatic and chromatic just noticeable differences (JND) thresholds by number of *Turdus* thrush species (% of species).


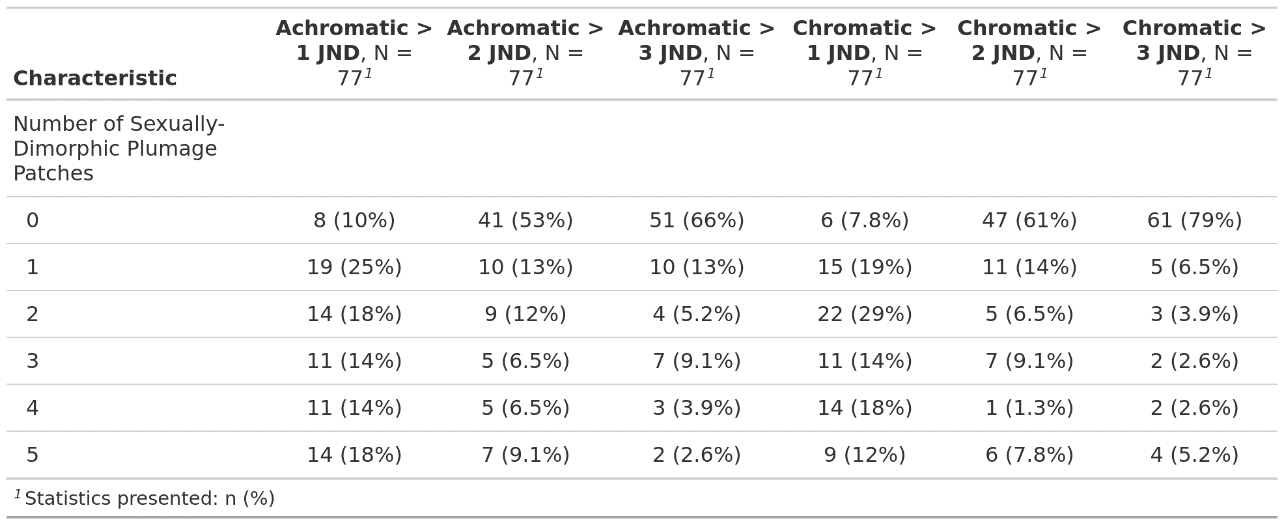


Table **S2**: Number of sexually-dimorphic plumage patches for separate achromatic and chromatic just noticeable differences (JND) thresholds by number of *Turdus* thrush species (% of species).


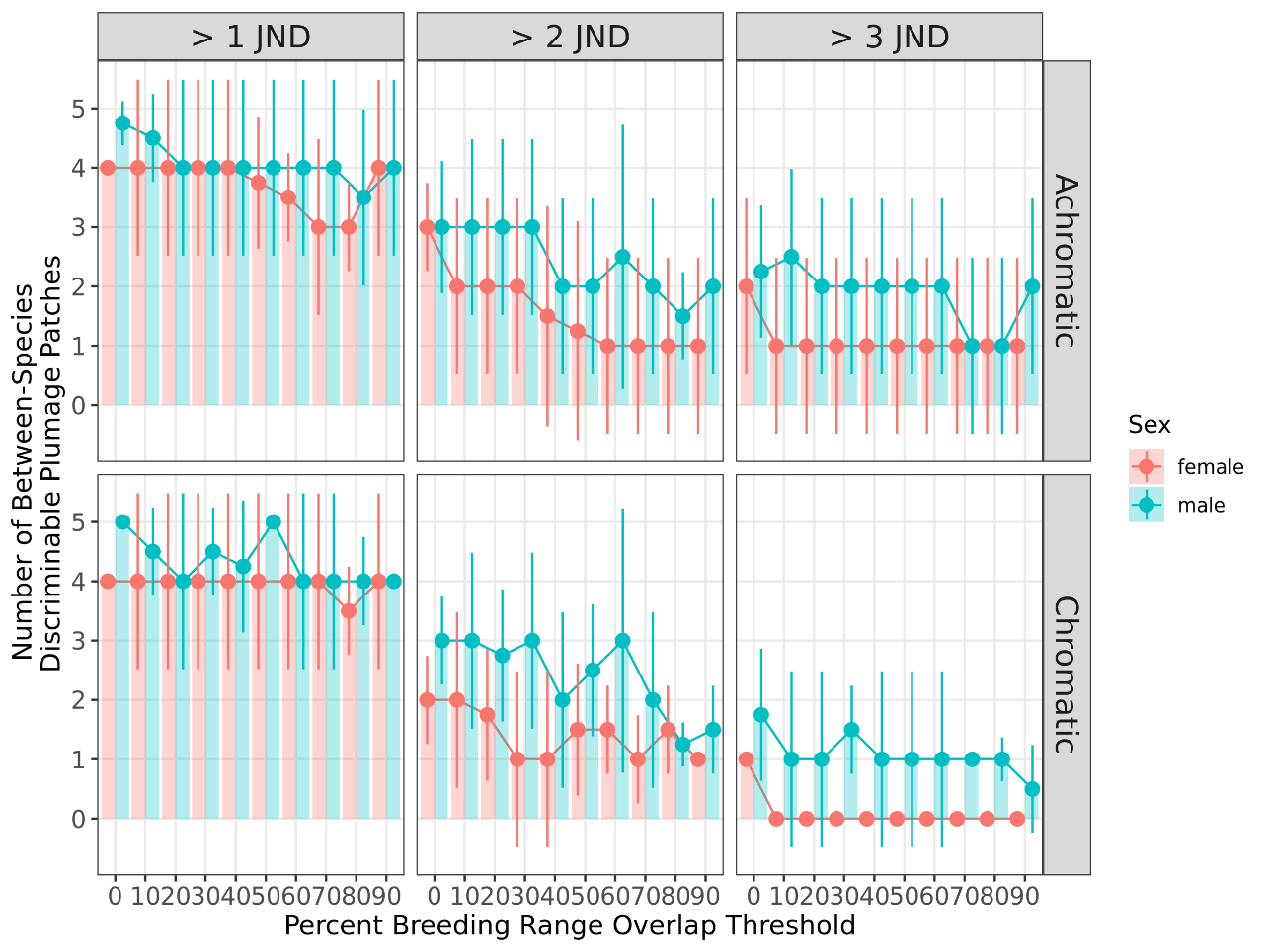


**Fig S1**: Median ± median absolute deviation of number of distinguishable plumage patches by just noticeable differences (JND) thresholds of 1, 2, and 3 between male and female *Turdus* thrush species in sympatry at various breeding range overlaps (percent).


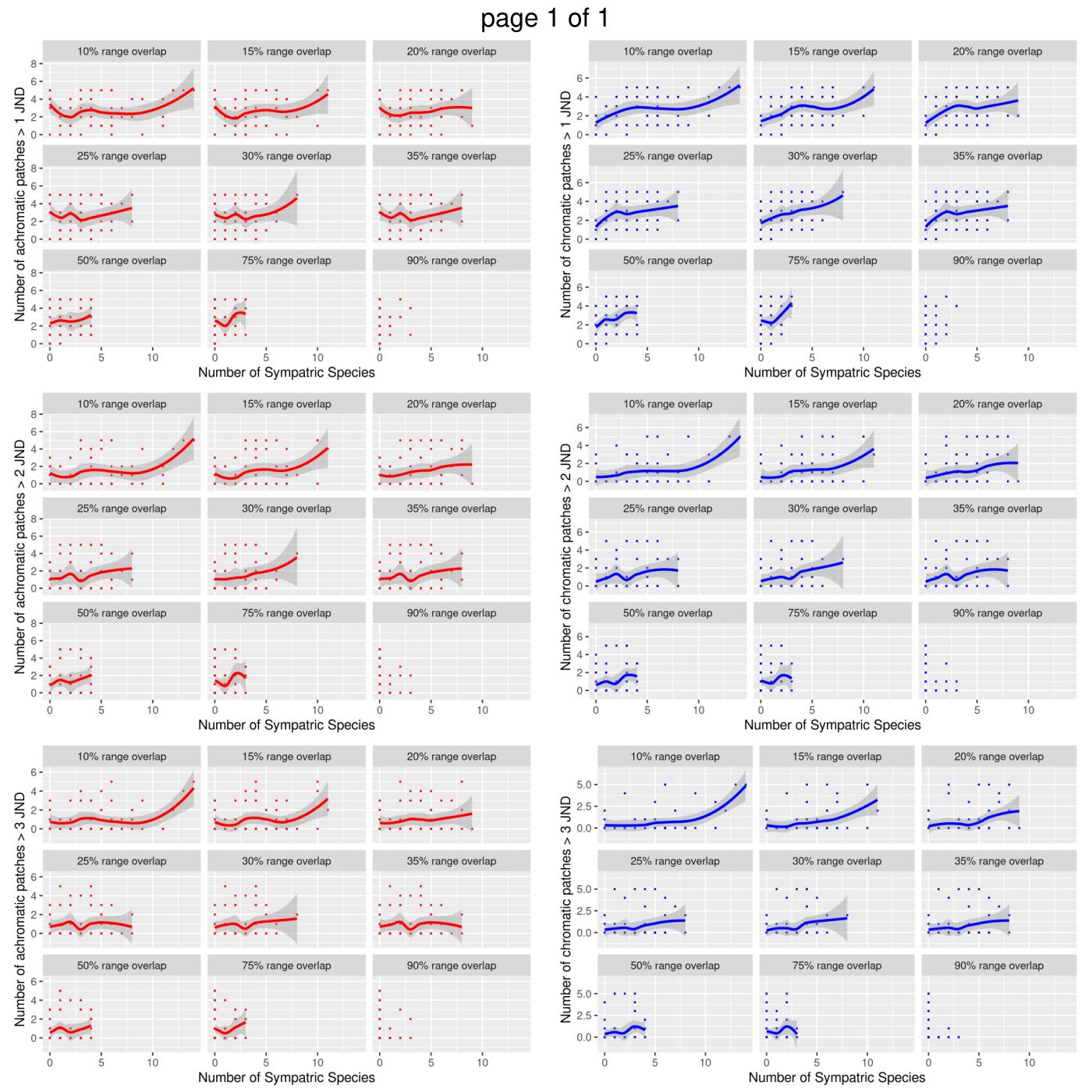


**Fig S2**: Number of sexually-dichromatic chromatic and achromatic plumage patches versus number of sympatric *Turdus* species, faceted by sympatry overlap thresholds (0-90%). Lines are Loess nonlinear regression fits with no correction for phylogenetic relatedness among species.


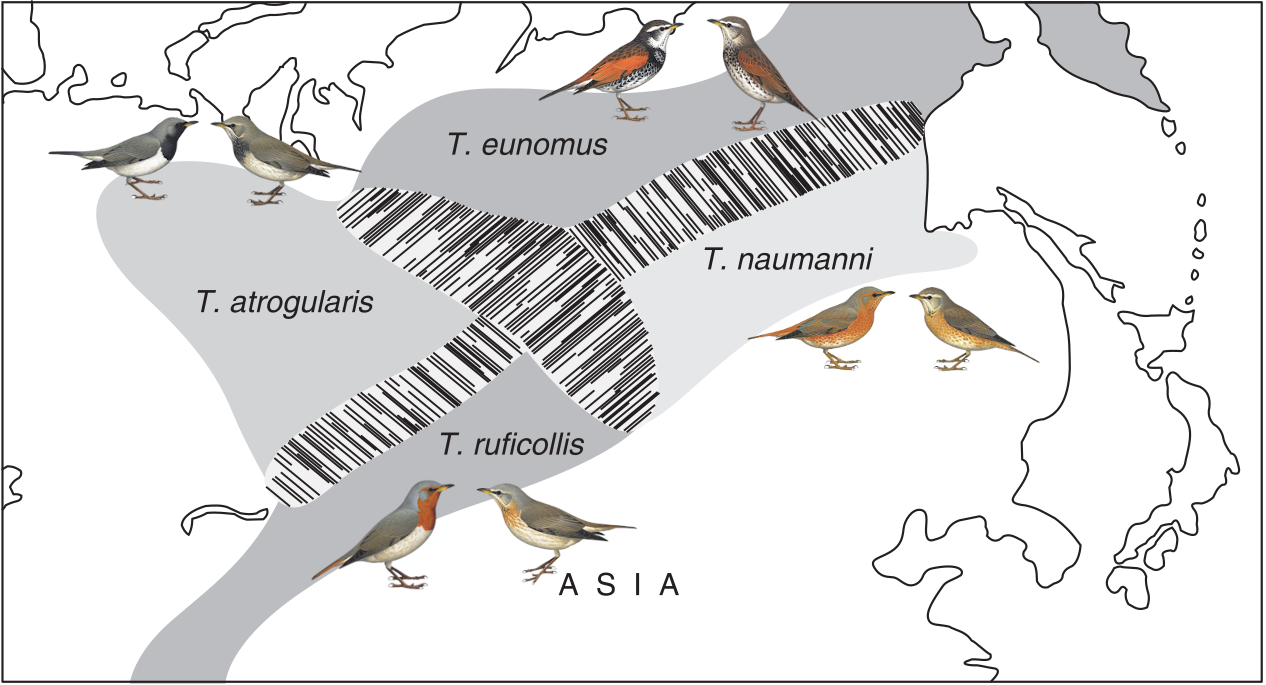


**Fig S3**: Four species hybrid zone in north-central Asia (*T.atrogularis*, *T.ruficollis*,*T.eunomus*, and *T.naumanni*). Map is from McCarthy (2006). Illustrations © HBW Alive/Lynx Edicions.

## References

1. McCarthy EM. 2006 *Handbook of avian hybrids of the world*. Oxford ; New York: Oxford University Press.
